# Y-chromosome genetic diversity of *Bos indicus* cattle in close proximity to the centre of domestication

**DOI:** 10.1038/s41598-020-66133-3

**Published:** 2020-06-19

**Authors:** Indrajit Ganguly, C. Jeevan, Sanjeev Singh, S. P. Dixit, Monika Sodhi, Ashish Ranjan, Suchit Kumar, Anurodh Sharma

**Affiliations:** 1 ICAR-National Bureau of Animal Genetic Resources, Karnal, 132001 Haryana India; 2 ICAR-National Dairy Research Institute, Karnal, 132001 Haryana India

**Keywords:** Genetic markers, Haplotypes, Population genetics

## Abstract

Y-chromosome genetic diversity in and around its domestication origin and a better understanding of indicine-specific microsatellite alleles are imperative concerns but less -targeted. We analysed Y-chromosome markers in 301 bulls representing 19 native Indian cattle (*Bos indicus*) and identified new alleles and haplotypes. Compared to other indicine studies, the high Y-haplotype diversity found in Indian cattle supports the hypothesis of greater genetic variability across the centre of origin decreasing along migratory routes with increasing distance. Hence, a considerable paternal genetic diversity of Indian cattle appears to have been lost in transboundary commercial indicine breeds. The Khillar and Gir are the most diversified populations where the first tends to be the well-differentiated traditional breed carrying strikingly distinct Y-lineages with typical BM861-158 bp allele, characteristics of taurine cattle, while retaining standard indicine lineages for all other markers. Geographical distribution found to be an unreliable predictor of parental variation, and Y-lineages seemed closely related to Indian breed function/utility. The comprehensive Y-chromosome information will be useful to examine the demographic expansion/spread of *Bos indicus* lineages from close proximity to the domestication centre across different countries worldwide and such diversity should be preserved through effective management and conservation programs.

## Introduction

The wild aurochs subspecies *B.p. primigenius* in Southwest Asia and *B.p. namadicus* in India are the ancestors of taurine (*Bos taurus*) and zebu (*Bos indicus*) cattle, respectively^1^. Around 2000 years after the taurine cattle domestication, zebu cattle were domesticated in the Indus Valley at the edge of the Indian Desert^2,3^. Later, pastoralist migrations from the centre of domestication introduced zebu cattle across the East Asia, southwestern Asia, Europe and African countries between 4000 and 1300 YBP, leading to admixture between zebu and taurine populations^4^. Over the past 150 years zebu cattle have reached America and Oceania, contributing to the growth of the developing economies^2,5,6^.

The identification of five single nucleotide polymorphisms (SNPs) has permitted the classification of worldwide extant cattle breeds into three Y-chromosome haplogroups, named Y1, Y2 and Y3^7^^.^ The Y chromosome diversity found in indicus cattle is represented by a single haplogroup (Y3) and variability within this haplogroup makes it possible to differentiate paternal lineages between Indian and Chinese cattle^8^ and to recognize the presence of Y3b haplotype family of Indian origin exclusively in West African Zebu animal^9^. The Y-specific microsatellite loci have been studied in several cattle breeds from different geographical areas^10–13^ or local breeds such as Ethiopian cattle^14^, Portuguese cattle^15^, Spanish cattle^16^ and Polish cattle^17^. The in-depth diversity analyses based on paternal lineages have allowed the identification of several Y-haplotypes within the major haplogroups. Generally, Y chromosome phylogenetic surveys of Indicine cattle include only a few breeds and have focused on taurine and zebuine crosses^18–20^. In this context, *Bos indicus* Y-chromosome diversity has not yet been extensively analyzed particularly close to their domestication centre.

India, where *Bos indicus* were domesticated, has rich cattle genetic resources with 50 well defined breeds (http://www.nbagr.res.in) classified according to their utility as dairy, draft and dual. Analysis of autosomal microsatellites (STRs) and mitochondrial DNA (mtDNA) sequences revealed a considerably high genetic diversity within Indian native cattle breeds^21–24^. However, Y-chromosome diversity of these native cattle breeds is yet to be explored. The analysis of Y chromosome variations will help to infer the origin and genetic relationships of Indian cattle breeds, generate comprehensive information about *Bos indicus* Y-chromosome haplotype diversity in close proximity to the centre of domestication and shed light over demographic expansion/spread of *B. indicus* lineages worldwide. Moreover, investigation of Y-chromosome haplotypes and patriline diversity could complement previous studies based on autosomal markers and help in defining conservation priorities. The aim of this study was therefore to investigate haplotype diversity and relationships among Indian native cattle breeds using a combination of SNPs and STRs specific to the non-recombinant region of the Y chromosome.

## Results

### Y-chromosome haplogroups

All the *B. indicus* bulls were found to be restricted to the Y3 haplogroup. PCR specific to USP9Y resulted a 362 bp fragment for Y1 and a 443 bp fragment for Y2 and Y3. The *Ssp* I enzyme cleaved the Y3 into two distinct fragments of 337 bp and 107 bp, allowing to differentiate Y3 from Y2 (Supplementary Fig. S2). Y2 had no cutting site for *Ssp* I.

### *Bos indicus* Y-chromosome STR alleles

All microsatellite markers except DDX3Y1 were polymorphic. Surprisingly, INRA189 and DDX3Y1 predominantly exhibited 90 bp and 249 bp allele, respectively across all the analysed *B. indicus* breeds. This observation was strikingly different from the widely published 88 bp and 245 bp standardized microsatellite allele size reported in *B. indicus*^13,15^. The expected allele size of INRA189 were 98 and 104 bp, respectively in HF and Jersey; whereas the standardized allele size of DDX3Y1 was 249 bp in HF and Jersey^13,15^. Changing electrophoresis mobility could be anticipated if the fluorescent dye is not the same. For confirming allele sizing and defining allele codes of above microsatellite loci to match previously published data, representative indicine samples were genotyped along with Holstein Friesian and Jersey bull samples (reference) in the same sequencing run. In the combine run of *B. indicus* and *B. taurus* samples, GeneMapper profile of present study revealed INRA189-100 and INRA189-106 bp alleles in Holstein Friesian and Jersey, respectively. On the other hand, DDX3Y1 amplification revealed a 249 bp allele in *B. indicus* and 253 bp allele in all the Holstein Friesian, Jersey and HF crossbred bulls. The GeneMapper profile above showed that the allele sizing was correct, but in the present study, the allele codes for INRA189 and DDX3Y1 STRs were off by 2 and 4 bp, respectively. This means that INRA189-90 allele in fact corresponds to previously published allele 88^25,26^, making it the most frequent allele for this marker in *B. indicus* cattle. Likewise, DDX3Y1 corresponds to previously published allele 245 and found to be monomorphic in all the indicine breeds. For the remaining loci, allele codes appeared to be matching published data. A total of 18 alleles were observed (Supplementary Table S2). The number of alleles per microsatellite varied from 1 (DDX3Y1) to 5 (UMN0307) with an average value of three. Seventeen breeds showed fixed *B. indicus* Y-specific microsatellite allele of 156 bp (BM861) (Supplementary Table S2). The UMN0103 STR showed two fragments in all the *Bos indicus* samples (Supplementary Table S2).

### Y-chromosome haplotype diversity

The combined analysis of three single nucleotide polymorphisms (ZFY9-C / T, DDX3Y1-C / T and UTY19-C/A) and five polymorphic Y-chromosome microsatellites (BM861, INRA189, UMN0103, UMN0307 and UMN0504) identified 17 *Bos indicus* haplotypes (H1Y3 to H15Y3; H18Y3 and H22Y3) in 301 bulls representing 19 Indian cattle breeds (Table 1). A common nomenclature was established by including Y3^7^ in our haplotype symbolization in order to make the comparison of public data available^7,13,15^, to reduce complexity and to highlight the correspondence between the identified alleles and the previously described ones (Supplementary Table S3). Among 13 rare haplotypes (9.94%), 11 haplotypes (7.28%) were limited to particular breeds and the remaining 2 haplotypes (H10Y3 and H13Y3), with a joint frequency of 2.66%, were restricted to two breeds each. Four haplotypes were shared across several breeds (H4Y3, H5Y3, H9Y3, and H18Y3) and had an overall frequency greater than 90% (Supplementary Fig. S1). They were recorded in 18, 30, 52 and 172 animals and shared by 5, 7, 7 and 13 breeds, respectively. Among the most frequent haplotypes, H18Y3 had the highest frequency (56. 81%) and was fixed in 3 breeds (Ongole, Sahiwal and Vechur). This haplotype also was at high frequency (>85%) in three other breeds (Gir, Rathi and Punganur). H9Y3 was the second most frequent (17.30%) haplotype observed in four breeds (Hariana, Kankrej, Mewati and Nagori) at frequencies>81% and at lower frequencies in three other breeds (Kherigarh, Tharparkar and Rathi). Haplotypes H5Y3 and H9Y3 were fixed in Kangayam and Hariana bulls, respectively. Haplotype diversity varied from 0.105 to 0.714 in breeds having more than three individuals and where more than one haplotype was observed (Table 1). Among these breeds, the most diverse were Malnad Gidda (H = 0.714 ± 0.052) and Khillar (H = 0.679 ± 0.102).

**Table 1:** Y-chromosome haplotype frequency and diversity of different Indian native cattle breeds (*Bos indicus*).

*Breeds/Lineages*	*N*	*Haplotype frequency (%)*																	*H*	*SD*
		H1Y3	H2Y3	H3Y3	H4Y3^*^	H5Y3	H6Y3	H7Y3	H8Y3	H9Y3^*,+^	H10Y3	H11Y3	H12Y3	H13Y3^*^	H14Y3	H15Y3	H18Y3^*,+^	H22Y3^*,+^		
Dangi	**3**		33.3		33.3	33.3													1.000	0.272
Gir	**56**											1.8	1.8			1.8	94.6		0.105	0.055
Hariana	**6**									100.0									0.000	0.000
Kangayam	**7**					100.0													0.000	0.000
Kankrej	**15**							6.7		86.7							6.7		0.257	0.141
Khillar	**20**	5.0		5.0		20.0	55.0				5.0				5.0			5.0	0.679	0.102
Krishna Valley	**3**				33.3	33.3											33.3		1.000	0.272
Malnad Gidda	**14**					28.6								35.7			35.7		0.714	0.052
Mewati	**16**									81.3							18.8		0.325	0.125
Nagori	**17**									88.2							11.8		0.221	0.12
Nimari	**16**				25.0	75.0													0.400	0.113
Ongole	**11**																100.0		0.000	0.000
Punganur	**7**								14.3								85.7		0.286	0.196
Rathi	**22**					4.5				4.5							90.9		0.177	0.106
Red Sindhi	**17**				64.7												35.3		0.485	0.078
Sahiwal	**52**																100.0		0.000	0.000
Tharparkar	**14**									21.4	7.1			7.1			64.3		0.571	0.132
Vechur	**3**																100.0		0.000	0.000
Kherigarh	**2**				50.0					50.0									1.000	0.500
Total	**301**																			
Overall haplotype diversity in *B. indicus* without subdivision into lineages																			**0.630**	**0.027**

Also reported earlier *^13^; ^+^^27^.

Pairwise F_ST_ values are presented in the Supplementary Table S4, and ranged from 0 to 1; where the 0 means no differentiation and 1 indicates highly differentiated populations. The F_ST_ value is the measure of potential frequency of heterozygotes if all members in a population are randomly mixed. Kangayam was the most and Tharparkar was the least differentiated breeds under study with average F_ST_ values (±standard deviation) of 0.735 ± 0.243 and 0.302 ± 0.183, respectively. Among dairy breeds, Sahiwal (0.646 ± 0.374) was the most differentiated and Thaparkar (0.302 ± 0.183) was the least. The most and least differentiated draft breeds were Kangayam (0.735 ± 0.243) and Malnad Gidda (0.347 ± 0.173), respectively. Among dual-purpose breeds, Hariana (0.640 ± 0.327) was the most diverse and Mewati (0.502 ± 0.276) was the least.

The pairwise F_ST_ represented in two dimensional space with multi-dimensional scaling resulted in 3 distinct clusters as shown in Fig. 2. The groups formed are as follows: *Group 1*: Gir, Sahiwal, Ongole, Punganur, Rathi, Vechur and Tharparkar; *Group 2*: Kankrej, Mewati, Nagori and Hariana; *Group 3*: Malnad Gidda, Krishna Valley, Red Sindhi, Kherigarh, Dangi, Khillar, Nimari and Kangayam. The dairy breeds clustered together along with dual purpose breed Ongole and miniature dairy breeds Vechur and Punganur. On the other hand, the draft breeds were clustered together along with Red Sindhi (group 3, Fig. 2). Whereas, the dual purpose breeds namely Kankrej, Mewati and Hariana as well as Nagori were grouped together on the bottom left away from other populations (group 2, Fig. 2). The analysis of molecular variance revealed that 36.13% and 63.87% of the total genetic variation is found within and among populations, respectively (Table 2). Table 2 shows the results of AMOVA for geographical and functional classification as well as MDS. While the first didn’t show significant differences among groups, the latter two resulted in significant differences of 41.01% and 49.56%, respectively (Table 2).

**Figure 1:**
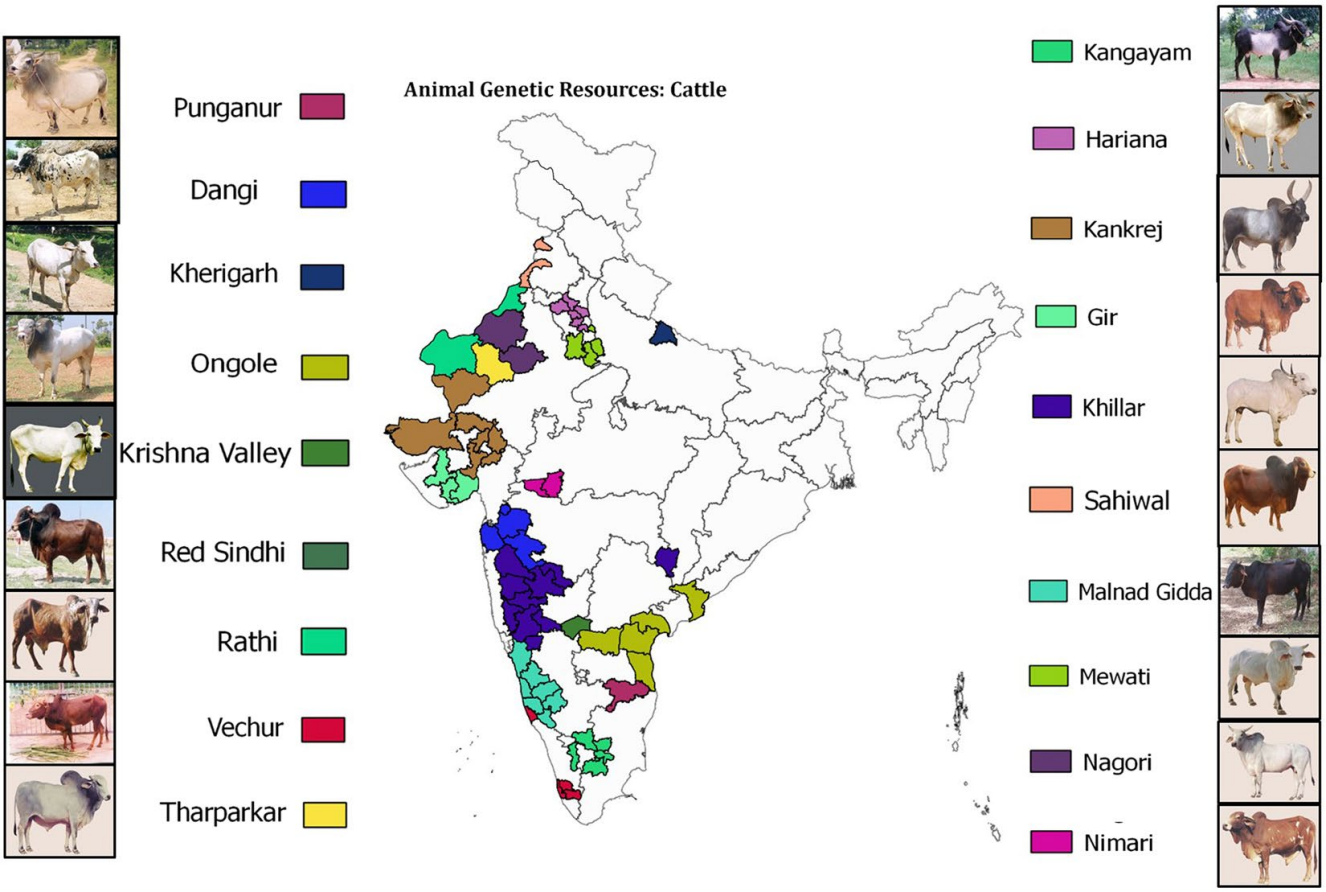
Distribution map of native Indian cattle breeds included in this study (created by QGIS 2.6 software; https://qgis.org/en/site/forusers/visualchangelog260/). The map has been ‘*Reproduced by permission of Surveyor General of India on behalf of Govt. of India under License No. BP15CDLA452. All rights reserved’*. For detail characteristics of each breed please visit Animal Genetic Resources of India (AGRI-IS) portal of our Institute ICAR-NBAGR, Karnal, Haryana, India (http://www.nbagr.res.in).

**Figure 2:**
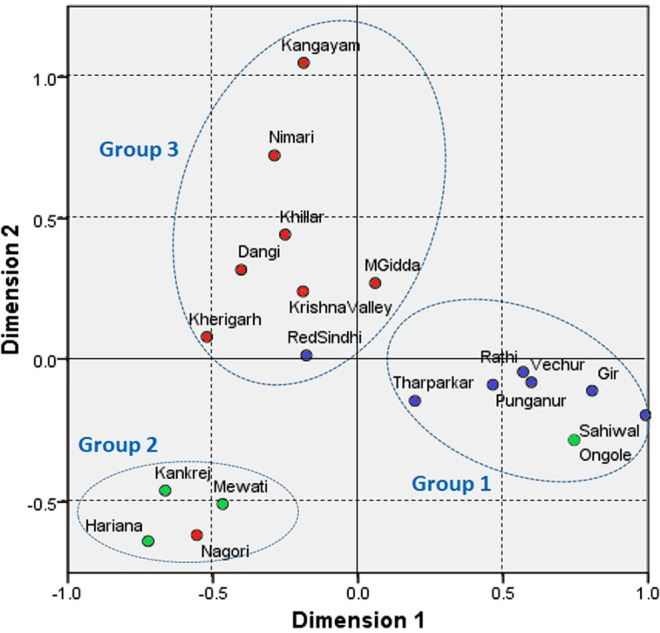
Genetic relationships between populations based on multidimensional scaling (MDS) by using F_ST_ distances. Blue circle-dairy breed; Red circle-draft breed; Green circle-dual Breed. M Gidda: Malnad Gidda.

**Table 2:** Analysis of molecular variance (AMOVA) for cattle Y chromosome haplotypes of Indian cattle breeds.

*Groups*	*N*	Variance components (%)			F statistics		
		*Among groups*	*Among breeds within group*	*Within breed*	*F*_*CT*_ *(p)*	*F*_*ST*_ *(p)*	*F*_*SC*_ *(p)*
**Geographical region**							
*North*	4	−13.68	75.96	37.72	−0.136 (>0.05)	0.622 (<0.0001)	0.668 (<0.0001)
*South*	8						
*West*	7						
*Overall*	19						
**Utility**							
*Dairy*	7	41.01	28.21	30.78	0.410 (<0.001)	0.692 (<0.0001)	0.478 (<0.0001)
*Dual*	4						
*Draught/Draft*	8						
*overall*	19						
**Without grouping**							
*All Bos indicus breeds under study*	19	-	63.87	36.13	-	0.638 (<0.0001)	-
**MDS groups**							
*Group 1*	7	49.56	20.46	29.98	0.495 (<0.0001)	0.700 (<0.0001)	0.405 (<0.0001)
*Group 2*	4						
*Group 3*	8						
*Overall*	19						

North: Mewati, Hariana, Kherigar, Sahiwal; West: Nimari, Kankrej, Tharparkar, Nagori, Red Sindhi, Rathi, Gir; South: Malnad Gidda, Dangi, Ongole, Khillar, Punganur, Vechur, Krishna Valley, Kangayam; Dairy: Gir, Sahiwal, Red Sindhi, Tharparkar, Punganur*, Vechur*, Rathi; Dual: Kankrej, Mewati, Ongole, Hariana; Draft: Malnad Gidda**, Nagori, Khillar, Kangayam, Krishna Valley, Kherigar, Nimari, Dangi. Group 1: Gir, Sahiwal, Punganur, Vechur, Rathi, Tharparkar, Ongole; Group 2: Kankrej, Mewati, Hariana, Nagori; Group 3: Malnad Gidda, Khillar, Dangi, Kherigarh, Krishna Valley, Red Sindhi, Kangayam, Nimari; *Miniature dairy breed; **Miniature draft breed; Average per lactation milk yield for dairy, dual and draft Indian cattle breeds are >1700 Kg, 600–900 Kg and <600 Kg, respectively (Source: Animal Genetic Resources of India -Agri IS portal of NBAGR; www. http://www.nbagr.res.in/).

**Figure 3:**
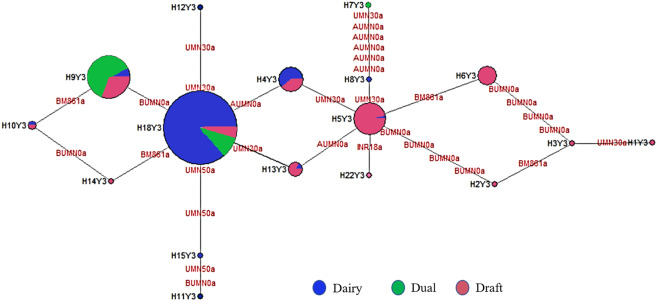
The MJ network representation of 17 Y-chromosome haplotypes identified in Indian native cattle breeds. The size of the circles correspond to haplotype frequencies. BM861: BM861a; INRA189: INR18a; UMN0103A: AUMN0a; UMN0103B: BUMN0a; UMN0307: UMN30a; UMN0504: UMN50a.

Relationships among Y chromosome haplotypes are shown in the median-joining network (Fig. 3). The network software generates median vectors representing haplotypes that are absent or not sampled. In the network of 17 indicine Y haplotypes, however, no median vector was found, and most haplotypes were either connected to core haplotypes H18Y3 or H5Y3. PCA analysis based on haplotype frequency revealed that 72.07% of the total variation is explained by the first four dimensions (Supplementary Fig. S3e). PC1 and PC2 explained 51.52% of total variation (Supplementary Fig. S3a); where Khillar and Gir cattle determine 75% of the variability (Supplementary Fig. S3b,d). Eight unique haplotypes of Khillar and Gir contributed the most to the overall genetic variation (indicated by red colours in Supplementary Fig. S3c). PCA analysis by removing most differentiated Khillar and Gir revealed distinct clustering of dairy, draft and dual purpose breeds (Supplementary Fig. S4).

## Discussion

### Finding new haplotypes in indicine cattle

As expected, all the haplotypes of *Bos indicus* breeds belong to Y3 haplogroup. In this study we could not find few rare Y3-haplotypes that have recently been reported in African cattle and American Creole breeds (Supplementary Table S3). *Bos indicus* gene pools mainly Ongole (Nellore), Kankrej (Guzerat) and Gir (Gyr) bulls reached the Americas 150 YBP^2^ and had been extensively used in European *B. taurus* cows, especially Creole breeds, followed by repeated backcrossing to *B. indicus* bulls. Therefore, as observed in our study, the most common Y-haplotypes (H18Y3 and H9Y3) are now widely present in international transboundary indicine breeds, Creole cattle and indigenous African cattle^13,27^ (Supplementary Table S3).

We find 11 private haplotypes among the Indian cattle, each restricted to a single breed. The Khillar carries as many as five unique haplotypes. Four haplotypes (H18Y3, H9Y3, H5Y3, and H4Y3), commonly seen in many *B. indicus* breeds, reflect shared ancestry/genetic signature. A similar pattern of frequency distribution, in which a few haplotypes represented higher frequency across breeds, has been reported in African^14^ and Portuguese^13,15^ cattle as well as in worldwide sheep^28^ breeds. Overall, the five breeds, namely Hariana, Kangayam, Ongole, Sahiwal, and Vechur, contain only one haplotype, and are therefore the least diverse. It should be noted here that the limited number of samples in some breeds affecting population analysis may be the reason for reduced haplotype diversity, especially in Hariana, Kangayam and Vechur. Despite the small sample sizes, each of the animals sampled in the Dangi, Krishna Valley and Kherigarh populations displayed distinct haplotypes resulting in a haplotype diversity of 1.00.

The new alleles identified in indicine bulls of Indian origin are BM861-158 bp; UMN0307-155 bp; UMN0504-148; UMN0504-150 bp; UMN0103-114/116 and UMN0103-124/124. The UMN0103 STR consistently revealed two alleles in all the bull samples, comparable to previous studies on *Bos indicus*^13,15,27,29^. Khillar appears to be a typical breed with very high frequency BM861-158 bp allele, a clear case of size homoplasy^30^. In addition, it does not even seem to be related to taurine animals, as all haplotypes are Y3.

The overall Y haplotype diversity (0.630 ± 0.027) observed in Indian cattle is reasonably higher than previously reported estimates in Polish cattle (0.037 ± 0.019)^17^, Lidia bovine breeds (0.42)^16^, Continental Europe (0.543 ± 0.026) and Zebu cattle (0.389 ± 0.084)^27^; but relatively lower than Ethiopian cattle (0.751 ± 0.015)^14^, Creoles (0.779 ± 0.019), Iberian breeds (0.712 ± 0.016) and cattle of Atlantic Islands (0.677 ± 0.037)^27^. Haplotype diversity within lineages as well as retention of allelic variants by various breeds act as a genetic reservoir. Nevertheless, *Bos indicus* Y-chromosome diversity is observed to be far less than that of humans (>0.89)^31^. This disparity is possibly due to the comparatively reduced effective male population size of cattle breeds. Use of artificial insemination (AI) in breeds like Sahiwal, Gir and Ongole as well as use of limited number of bulls in other breeds (Vechur and Hariana) may be associated with their reduced Y-haplotype diversity. Restricted use of males^32^ as well as extensive use of AI^15^ have been reported to be the key reasons for reducing the number of patrilines in horse and cattle, respectively, based on Y-chromosome polymorphism.

The present study demonstrates the existence of high Y-chromosome diversity in *Bos indicus*. It supports the expectation of greater genetic diversity surrounding centres of origin which declines with distance from the primary site of domestication^33^. Comparatively lower Y-chromosome diversity has recently been reported in transboundary indicine cattle^13^. The autosomal microsatellite based diversity analyses have also revealed high allelic diversity and heterozygosity in Indian native cattle breeds^23,24,34^. Overall, the diversity in Indian cattle was found to be higher than that of their European counterparts^35^, and might be attributed to lack of artificial selection pressure, relatively larger effective population size, as well as proximity to the domestication centre especially in the case of *Bos indicus*. Genome-wide survey of SNPs also revealed a much larger ancestral population (pre-domestication *Ne*) of indicine breeds originated in Southern Asia compared to modern taurine cattle^35^.

### There is some degree of genetic differentiation between breeds

The average F_ST_ value over all *B. indicus* breeds (0.490 ± 0.106) shows that a considerable amount of variation is explained by breed differences. The present analysis finds no geographical grouping of Indian cattle breeds. Similar trends have also been recorded in Portuguese cattle breeds^15^, with contrasting results in dog^36^, sheep^28^ and human^31^. However, contrary to the present findings, geographical clustering of Y chromosome variability has been described for several species showing male-mediated dispersal where differences among population groups are further augmented by genetic drift^28,31,36,37^. Grouping of breeds in the present study tends to follow the division of dairy, draft or dual. An explanation for the clustering would be their common paternal roots. The presence of haplotype H5Y3 with a comparatively higher frequency in Kangayam and Nimari positions them far apart from other breeds in the draft category (Fig. 2). Without median vector the haplotype network indicated a simple and conservative evolutionary patterns across the indicine breeds (Fig. 3). Altogether, MDS, AMOVA, PCA and MJ network analyses demonstrated that geographical distribution is not a reliable predictor of paternal variation, and Y-lineages appear to be more related to function of the Indian cattle breeds. The clustering of Indian cattle breeds based on genome-wide runs of homozygosity further supports the view^38^.

## Conclusion

In summary, native *B. indicus* cattle from India retain high levels of paternal genetic diversity which appears to have been lost in transboundary commercial indicine cattle. Such diversity should be maintained through management and conservation plans. Moreover, comprehensive information pertaining to Y-chromosome diversity of *Bos indicus* described here can be used to investigate the demographic expansion/spread of *Bos indicus* lineages throughout different countries from the close proximity to the domestication centre.

## Methods

### Sample collection and DNA isolation

A total of 301 random bull samples comprising 19 native cattle breeds (*Bos indicus*) of India were included (Fig. 1). They represented various agro-climatic zones and were of different utility purposes (dairy/draft/dual). Frozen semen samples were obtained from different livestock semen stations located at Hissar (Haryana), Rishikesh (Uttarakhand), Patan (Gujrat), Jagadhri (Haryana), Bhopal (MP) and Gene Bank repository of ICAR-NBAGR, Karnal (Haryana). Jersey, Holstein Friesian and Holstein Friesian crossbred bulls were included for screening Haplogroups and confirmation of microsatellite alleles. Two Sahiwal cow DNA samples were also incorporated in all assays to validate Y-specificity of primers. Genomic DNA was extracted from frozen semen straws as follows:

### Prewash of sperm

For every bull, 0.5 ml semen sample was collected from two frozen semen straws (2 × 0.25 ml) in a 2.0 ml eppendorf tube after thawing. Sample was washed with 70% ethanol and centrifuged at 13000 rpm for 5 min. Supernatant was discarded without disturbing the cell pellet.

### Cell lysis and protein digestion

750 µl of lysis buffer (10 mM Tris HCL, 25 mM EDTA, 1% SDS, 75 mM NaCl) was added to the cell pellet followed by addition of 3.75 µl Triton X-100, 31.5 µl of DTT and 15 µl proteinase-K (20 mg/ml). The contents were mixed well for 5 min and kept at 50 °C in a water bath overnight. Later, digested content was cooled down to room temperature and approximately an equal volume of Phenol: Chloroform: Isoamyl alcohol (650 µl of PCI per 500 µl of sample), was added. The content was mixed gently and centrifuged at 13000 rpm for 5 min. Aqueous phase was separated and transferred to a clean eppendorf tube (2.0 ml) followed by addition of 1.0 ml Chloroform and Isoamyl alcohol (24:1). The contents were mixed gently and centrifuged at 13000 rpm for 5 min. The uppermost aqueous phase containing DNA was separated without disturbing the middle and lower phases.

### DNA precipitation

1/10^th^ volume of sodium acetate was added followed by addition of equal volume of chilled absolute ethanol, to precipitate the DNA. The precipitated DNA pellet was washed 2–3 times with 70% ethanol by centrifugation at 5000 rpm for 5 min. Discarded the supernatant and air dried the pellet carefully avoiding over dryness while leaving no traces of ethanol. Later, the pellet was dissolved in 100 µl Milli-Q water. The concentration of DNA was checked with Nanodrop spectrophotometer (Nanodrop Technologies, Wilmington, MA, USA). The quality of DNA was verified on 0.8% agarose gels.

### Genotyping of Y-chromosome SNPs and microsatellite markers

Three single nucleotide polymorphisms (ZFY9-C/T, DDX3Y1-C/T and UTY19-C/A)^7^, were analysed by allele-specific PCR (AS-PCR) protocols^39,40^. ZFY9 (C/T) and DDX3Y1 (C/T) were genotyped to differentiate zebu (Y3) and taurine specific (Y1 and Y2) Y-haplogroups; whereas, UTY19 (C/A) was analysed to distinguish between Y1 and Y2. Y-haplogroups were further confirmed by PCR-RFLP of USP9Y marker^41^ using newly designed primers and sequencing representative samples. Based on GeneBank sequences (JF923763, Y1; JF923764, Y2 and JF923765, Y3), new primers were designed. (a) Forward primer USP9YF: 5′ GGG GCT TAG AGT GCT CCA GT 3′, (b) Reverse primer USP9YR: 5′ ACA GCT CCT CAA AAC CAG AAT 3′. The standardized PCR protocol was as follows: Initial denaturation at 95 °C for 5 min, followed by 35 cycles of denaturation at 94 °C for 30 s, annealing at 60 °C for 30 s, extension at 72 °C for 30 s and the final extension at 72 °C for 10 min. PCR products of 10 µL were subjected to restriction endonuclease enzyme digestion by *Ssp* I enzyme (5U per reaction) at 37 °C for 3 hours. The products (5 µL) were later subjected to agarose gel (2%) electrophoresis and the samples were grouped based on the bands obtained.

Six STRs (DDX3Y1, BM861, INRA189, UMN0103, UMN0307 and UMN0504), specific to the non-recombining region of Y-chromosome, were genotyped. Detailed information of primers is presented in Supplementary Table S1. The 5′ ends of the forward primers were labelled with either VIC, NED, or FAM dyes. The PCR conditions were standardized for all of the primer pairs selected for the study. PCR reaction was performed for each microsatellite separately by using specific set of primers in a 15 µl final reaction volume. The reaction mixture was prepared by adding 1.5 µl of 10X buffer, 1.5 mM of MgCl_2_, 200 µM dNTPs, 0.25 µM of each forward and reverse primer, 1U of Taq DNA polymerase and approximately 50 ng of genomic DNA in a total volume of 15 µl by adding molecular grade water. The standardized thermocycling protocol for each microsatellite used to screen the haplotypes was as follows: initial denaturation at 95 °C for 5 min, following 35 cycles of denaturation at 94 °C for 30 s, annealing at 58/62 °C (primer specific, Supplementary Table S1), extension at 72 °C for 30 s and final extension at 72 °C for 10 min. The PCR products were visualized on 2% agarose gels after ethidium bromide staining (0.5 µg/ml). Post-PCR multiplexing was used to genotype 2 or 4 loci simultaneously (Supplementary Table S1). Genotyping was carried out on an automated ABI-3100 DNA sequencer (Applied Biosystems, USA), with GeneScan–500 LIZ as the internal lane size standard (Thermo Fisher Scientific). Allele sizing was done by using GeneMapper software 5 (Thermofisher Scientific). The allele data thus generated were used for subsequent statistical analyses. DNA samples from two cows were included in all the assays to validate Y-specificity of primers. To confirm allele size of microsatellite loci, specifically INRA189, BM861 and DDX3Y1, representative *B. indicus* samples carrying different alleles were run together with Holstein Friesian and Jersey bulls in single sequencing run. Besides, a number of Holstein Friesian (n = 49), Jersey (n = 25) and HF crossbred (n = 10) bulls were genotyped to verify allele sizing and correct allele code assignment.

### Statistical analysis

Microsatellite data was analysed for estimation of haplotype frequencies in different breeds using GenAlEx 6.5^42^. Frequencies of Y chromosome haplotypes were then used to compute the haplotype diversity (H) for each breed and pairwise F_ST_ genetic distances with 10,000 permutations at 5% level of significance using ARLEQUIN v3.5^43^. The pairwise F_ST_ values were represented in two-dimensional space with multi-dimensional scaling in the SPSS version 24.0 software package. Analysis of Molecular Variance (AMOVA) was done with ARLEQUIN v3.5 without subdivision into groups, as well as partitioning the total genetic variation through grouping the native breeds as follows: by geographic region as Northern (Mewati, Hariana, Kherigar, Sahiwal), Southern (Malnad Gidda, Dangi, Ongole, Khillar, Punganur, Vechur, Krishna Valley, Kangayam) and Western (Nimari, Kankrej, Tharparkar, Nagori, Red Sindhi, Rathi, Gir); by breed utility characteristics such as dairy (Gir, Sahiwal, Red Sindhi, Tharparkar, Punganur, Vechur, Rathi), draft (Malnad Gidda, Nagori, Khillar, Kangayam, Krishna Valley, Kherigar, Nimari, Dangi) and dual (Kankrej, Mewati, Ongole, Hariana); and by MDS grouping (Group 1: Gir, Sahiwal, Punganur, Vechur, Rathi, Tharparkar, Ongole; Group 2: Kankrej, Mewati, Hariana, Nagori; Group 3: Malnad Gidda, Khillar, Dangi, Kherigarh, Krishna Valley, Red Sindhi, Kangayam, Nimari). Vechur and Punganur are miniature dairy cattle breeds of India (http://www.nbagr.res.in). Significance levels for the estimated fixation indices were obtained by comparison of the actual values with 10,000 permutations. Phylogenetic relationships among haplotypes were investigated using a median-joining network (M-J) implemented in NETWORK v4.2.0.1 (Fluxus Technology Ltd., Suffolk, England)^44^. Reduced-median joining analysis was performed first and the output was then used as input for the Median-Joining calculations. As in previously published studies, UMN0103 was divided into A & B because of having two alleles/loci with variable sizes specific to *Bos indicus*. Locus specific weights were given proportional to their within-breed variance component. Accordingly, polymorphic loci with highest variance were given the lowest weight^14^. Such weights were intended to account for the differences in genetic variation of different microsatellite loci^12^. Hence, STRs BM861, INRA189, UMN0103A, UMN0103B, UMN0307, UMN0504 were given weights of 7, 9, 5, 4, 6 and 8, respectively. However, for major haplotypes the proportional change in weights allocated to the loci showed no noticeable change in the network structure. The principal components analysis was performed by using Y-haplotype frequencies. The R package “factoextra” was used to compute the eigenvalues and contribution of the PCs to the total genetic variation. The different graphs and plots were generated representing the contribution of the loci and individuals to the total genetic variation.

### Ethics statement

Semen samples, routinely collected by various semen stations in the country following standard procedures were incorporated. The frozen semen straws thus obtained were not directly related to our research project. Cow blood samples were obtained with written informed consent from the owner during veterinary inspections under the official health care program (fertility camp) by a qualified veterinarian in compliance with the relevant guidelines issued by the Committee for the Purpose of Control and Supervision of Experiments on Animals (CPCSEA; http://cpcsea.nic.in/WriteReadData/userfiles/file/Compendium%20of%20CPCSEA.pdf) and approved by the Institutional Animal Ethics Committee (IAEC) of ICAR-National Bureau of Animal Genetics Resources (ICAR-NBAGR), Karnal.

## Supplementary information

Supplementary information.

Supplementary information 2.

## Data Availability

The Supplementary Table S5 (xlsx file) includes all sample genotypes pertaining to the microsatellite loci used in the study.
