# Supplementary material for: Y-chromosome genetic diversity of Bos indicus cattle in close proximity to the centre of domestication
Source: Sci Rep. 2020 Jun 19;10:9992. doi: 10.1038/s41598-020-66133-3 (PMC7305206; doi:10.1038/s41598-020-66133-3)
Supplement: Supplementary file 1 — Supplementary information. [file 41598_2020_66133_MOESM1_ESM.pdf]

Supplementary information for

Y-chromosome genetic diversity of *Bos indicus* cattle in close proximity to the centre of domestication

Indrajit Ganguly[1]*, C Jeevan[2], Sanjeev Singh[1], S P Dixit[1], Monika Sodhi[1], Ashish Ranjan[2], Suchit Kumar[2] & Aurodh Sharma[1]

Affiliations:

[1]ICAR-National Bureau of Animal Genetic Resources, Karnal, 132001, Haryana, India.

[2]ICAR-National Dairy Research Institute, Karnal, 132001, Haryana, India.

*To whom correspondence and request should be addressed (e-mail: indrajit.ganguly@icar.gov.in)

The PDF file includes:

Supplementary Table S1: Primer sequences, Ta, fluorescence label and their allelic range, repeat motif and ratio of multiplexing for Y-chromosome STR loci.

Supplementary Table S2: Y-chromosome microsatellite allele frequencies of 19 Indian cattle breeds.

Supplementary Table S3: Y-haplotypes defined by 6 Y-chromosome microsatellites and their classification into haplogroup (s) by screening Y-specific markers ZFY9 (C/T), DDX3Y1 (C/T) and UTY19 (C/A).

Supplementary Table S4: Pairwise F_{ST} values (values in bold are statistically significant at level 5%) and average pairwise F_{ST} per breed (± standard deviation).

Supplementary Fig. S1: Major Y-chromosome haplotypes identified in Indian native cattle breeds.

Supplementary Fig. S2: Analysis of USP9Y marker to differentiate Y1, Y2 and Y3 haplogroups using *Ssp* I PCR-RFLP.

Supplementary Fig. S3: PCA analysis based on Y-chromosome haplotype frequencies of all breeds.

Supplementary Fig. S4: PCA analysis based on Y-chromosome haplotype frequencies of remaining breeds after removal of Gir and Khillar.

Supplementary Table S1: Primer sequences, Ta, fluorescence label and their allelic range, repeat motif and ratio of multiplexing for Y chromosome STR loci.

Sl.No.	Locus Name/ Primer name	Primer sequence (5'-3')	Label	Ta* (°C)	Reference	Allelic range	Repeat motif	Ratio of Multiplexing
1	INRA189-F	TACACGCATGTCCTTGTTTCGG	Fam	58	Kappes *et al.* (1997)[1]	68–124	$(TG)_{22}$	1:2
	INRA189-R	CTCTGCATCTGTCCTGGACTGG	-					
2	BM861-F	TTGAGCCACCTGGAAAGC	Ned	62	Bishop *et al.* (1994)[2]	144-158	$(GT)_6$ C $(TG)_{10}$	
	BM861-R	CAAGCGGTTGGTTCAGATG	-					
3	DDX3Y1STR-F	TGAACCACTAGGGAGGTCATC	Fam	58	Götherström *et al.* (2005)[3]	249	$(TA)_9$ $(TC)_9$	1 : 1 : 1.5 : 2
	DDX3Y1STR-R	TTCCAATTTAGCTGTGGTTATCTG	-					
4	UMN0103-F	ACACAGAGTATTCACCTGAG	Fam	58	Liu *et al.* (2003)[4]	124–136	$(CA)_{22}$	
	UMN0103-R	ATTTACCTGGGTCAAAGCAC	-					
5	UMN0307-F	GATACAGCTGAGTGACTAAC	Vic	58	Liu *et al.* (2003)[4]	101–162	$(CA)_{18}$	
	UMN0307-R	GTGCAGACATCTGAGCTGTG	-					
6	UMN0504-F	AGGCCATCTGCATAGTGAAG	Ned	58	Liu *et al.* (2003)[4]	106–144	$(CT)_2$ $GT(CT)_3$ $(GT)_2$	
	UMN0504-R	TGCTGGACTGCTCATCTCTG	-					

*Ta used in present study

Supplementary Table S2: Y- chromosome microsatellite allele frequencies of 19 Indian cattle breeds.

		BREED																		
**Marker**	**Length (bp)**	**DAG**	**GIR**	**HAR**	**KGM**	**KNK**	**KHL**	**KV**	**MG**	**MWT**	**NGR**	**NMR**	**ONG**	**PNG**	**RAT**	**RSD**	**SW**	**TP**	**VC**	**KHG**
		**3**	**56**	**6**	**7**	**15**	**20**	**3**	**14**	**16**	**17**	**16**	**11**	**7**	**22**	**17**	**52**	**14**	**3**	**2**
**UMN0103**	**114,116**	0.33	0	0	0	0	0.1	0	0	0	0	0	0	0	0	0	0	0	0	0
	**114,124**	0.67	0	0	1	0	0.8	0.67	0.29	0	0	1	0	0.14	0.05	0.65	0	0	0	0.5
	**116,122**	0	0.02	1	0	0.87	0.05	0	0	0.81	0.88	0	0	0	0.05	0	0	0.29	0	0.5
	**116,124**	0	0.98	0	0	0.07	0.05	0.333	0.71	0.19	0.12	0	1	0.86	0.91	0.35	1	0.71	1	0
	**124,124**	0	0	0	0	0.07	0	0	0	0	0	0	0	0	0	0	0	0	0	0
**UMN0307**	**145**	0	0.02	0	0	0	0	0	0	0	0	0	0	0	0	0	0	0	0	0
	**149**	0.33	0.98	1	0	0.93	0.15	0.67	0.36	1	1	0.25	1	0.86	0.96	1	1	0.93	1	1
	**151**	0.67	0	0	1	0	0.85	0.33	0.64	0	0	0.75	0	0	0.05	0	0	0.07	0	0
	**153**	0	0	0	0	0	0	0	0	0	0	0	0	0.14	0	0	0	0	0	0
	**155**	0	0	0	0	0.07	0	0	0	0	0	0	0	0	0	0	0	0	0	0
**UMN0504**	**144**	1	0.96	1	1	1	1	1	1	1	1	1	1	1	1	1	1	1	1	1
	**148**	0	0.02	0	0	0	0	0	0	0	0	0	0	0	0	0	0	0	0	0
	**150**	0	0.02	0	0	0	0	0	0	0	0	0	0	0	0	0	0	0	0	0
**BM861**	**156**	1	1	1	1	1	0.2	1	1	1	1	1	1	1	1	1	1	0.93	1	1
	**158**	0	0	0	0	0	0.8	0	0	0	0	0	0	0	0	0	0	0.07	0	0
**INRA189**	**88**	1	1	1	1	1	0.95	1	1	1	1	1	1	1	1	1	1	1	1	1
	**90**	0	0	0	0	0	0.05	0	0	0	0	0	0	0	0	0	0	0	0	0
**DDX3Y1**	**245**	1	1	1	1	1	1	1	1	1	1	1	1	1	1	1	1	1	1	1

DNG: Dangi, GIR: Gir, HR: Hariana, KGM: Kangayam, KNK: Kankrej, KHL: Khillar, KV: Krishna Valley, MG: Malnad Gidda, MWT: Mewati, NGR: Nagori, NMR: Nimari, ONG: Ongole, PNG: Punganur, RAT: Rathi, RSD: Red Sindhi, SW: Sahiwal, TP: Tharparkar, VC: Vechur; KHG: Kherigarh

Supplementary Table S3: Y -haplotypes defined by 6 Y-chromosome microsatellites and their classification into haplogroup (s) by screening Y-specific markers ZFY9 (C/T), DDX3Y1 (C/T) and UTY19 (C/A).

Haplotype (*Common nomenclature*)	STR(s)							
	DDX3Y1	BM861	INRA189	UMN0103	UMN0307	UMN0504	Ginja *et al*. (2019)[5]	Ginja *et al*. (2010)[6]
H1Y3	245	158	88	114/116	149	144	-	-
H2Y3	245	156	88	114/116	151	144	-	-
H3Y3	245	158	88	114/116	151	144	-	-
H4Y3*+	245	156	88	114/124	149	144	H1	-
H5Y3	245	156	88	114/124	151	144	-	-
H6Y3	245	158	88	114/124	151	144	-	-
H7Y3	245	156	88	124/124	155	144	-	-
H8Y3	245	156	88	114/124	153	144	-	-
**H9Y3*+**	245	156	88	116/122	149	144	H2	**H9Y3**
H10Y3	245	158	88	116/122	149	144	-	-
H11Y3	245	156	88	116/122	149	150	-	-
H12Y3	245	156	88	116/124	145	144	-	-
H13Y3*+	245	156	88	116/124	151	144	H4	-
H14Y3	245	158	88	116/124	149	144	-	-
H15Y3	245	156	88	116/124	149	148	-	-
**H18Y3*+**	245	156	88	116/124	149	144	H3	**H18Y3**
H19Y3**	245	156	88	118/122	149	144	H7	H19Y3
H20Y3**	245	156	88	118/124	149	144	H8	H20Y3
H21Y3**	245	156	88	122/130	149	144	H9	H21Y3
H22Y3*+	245	156	90	114/124	151	144	H10	H22Y3
H16Y3##	245	156	88	116/124	153	-	H5	-
H17Y3##	245	156	88	116/126	149	-	H6	-
H23Y3##	245	156	90	114/126	151	-	H11	-
H24Y3##	245	156	92	116/124	149	-	H12	-

** Reported in American Creole cattle[5,6] but not observed in the present study; *+ Observed in present study and also reported earlier[5,6]; ## Observed only in African Zebu[5]; Bold indicates most common indicine Y-haplotype; Principal haplogroup-Y3 has been confirmed by PCR-RFLP of USP9Y marker.

Supplementary Table S4: Pairwise F_{ST} values (values in bold are statistically significant at level 5%) and average pairwise F_{ST} per breed (± standard deviation).

BREED	DNG	GIR	HR	KGM	KNK	KHL	KV	MG	MWT	NGR	NMR	ONG	PNG	RAT	RSD	SW	TP	VC	KHG
**DNG**																			
**GIR**	0.812																		
**HR**	0.676	0.907																	
**KGM**	0.523	0.908	1.000																
**KNK**	0.577	0.848	-0.049	0.827															
**KHI**	0.166	0.710	0.535	0.453	0.514														
**KV**	-0.286	0.669	0.676	0.523	0.565	0.166													
**MG**	0.104	0.548	0.541	0.403	0.507	0.262	0.044												
**MWT**	0.518	0.801	0.029	0.780	0.000	0.487	0.479	0.451											
**NGR**	0.621	0.847	0.000	0.846	0.000	0.539	0.601	0.528	0.000										
**NMR**	0.101	0.817	0.722	0.104	0.670	0.357	0.101	0.296	0.638	0.692									
**ONG**	0.791	-0.034	1.000	1.000	0.842	0.592	0.641	0.408	0.768	0.850	0.768								
**PNG**	0.483	0.037	0.846	0.857	0.717	0.460	0.242	0.235	0.629	0.731	0.640	0.069							
**RAT**	0.676	-0.006	0.856	0.861	0.766	0.576	0.465	0.369	0.749	0.803	0.715	-0.169	-0.027						
**RSD**	0.176	0.665	0.664	0.676	0.615	0.414	-0.003	0.319	0.564	0.632	0.471	0.570	0.413	0.530					
**SW**	0.946	0.016	1.000	1.000	0.936	0.785	0.896	0.677	0.899	0.936	0.899	0.000	0.374	0.080	0.781				
**TP**	0.304	0.253	0.533	0.639	0.467	0.368	0.087	0.137	0.370	0.475	0.518	0.169	0.020	0.100	0.319	0.427			
**VC**	0.500	-0.846	1.000	1.000	0.773	0.477	0.250	0.231	0.678	0.791	0.678	0.000	-0.167	-0.178	0.436	0.000	-0.017		
**KHG**	-0.200	0.846	0.538	0.831	0.264	0.254	-0.200	0.221	0.194	0.329	0.418	0.888	0.565	0.731	0.095	0.975	0.255	0.647	
**AVERAGE F_{ST}**	0.443	0.538	0.640	0.735	0.549	0.451	0.353	0.347	0.502	0.568	0.534	0.520	0.407	0.460	0.463	0.646	0.302	0.415	0.447
**SD**	0.286	0.359	0.327	0.243	0.293	0.165	0.283	0.173	0.276	0.295	0.246	0.369	0.291	0.329	0.209	0.374	0.183	0.354	0.306

DNG: Dangi, GIR: Gir, HR: Hariana, KGM: Kangayam, KNK: Kankrej, KHL: Khillar, KV: Krishna Valley, MG: Malnad Gidda, MWT: Mewati, NGR: Nagori, NMR: Nimari, ONG: Ongole, PNG: Punganur, RAT: Rathi, RSD: Red Sindhi, SW: Sahiwal, TP: Tharparkar, VC: Vechur; KHG: Kherigarh

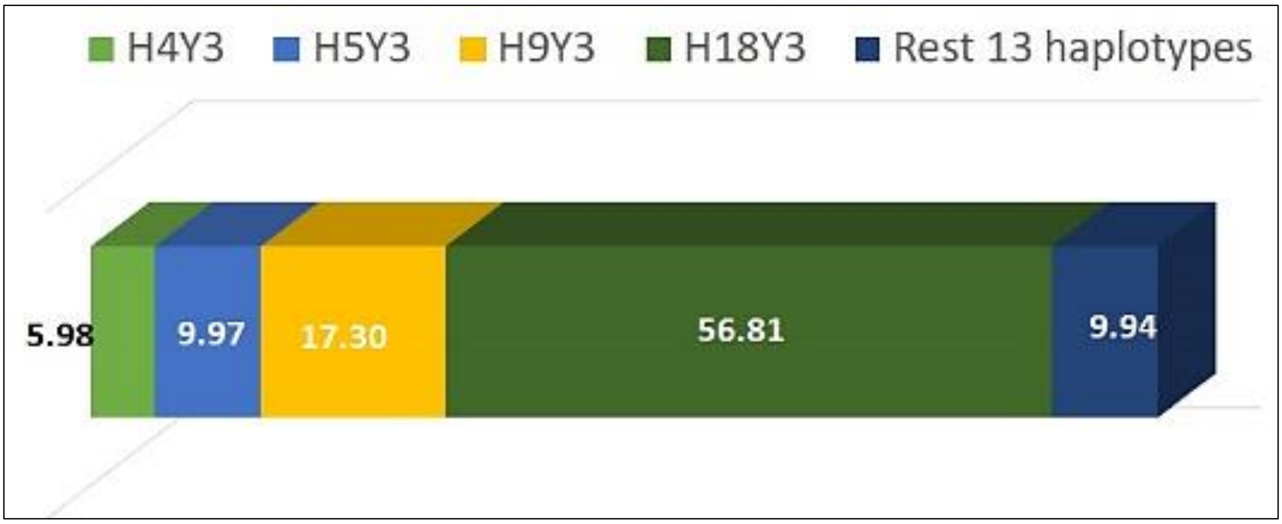

Supplementary Fig. S1: Major Y-chromosome haplotypes identified in Indian native cattle breeds.

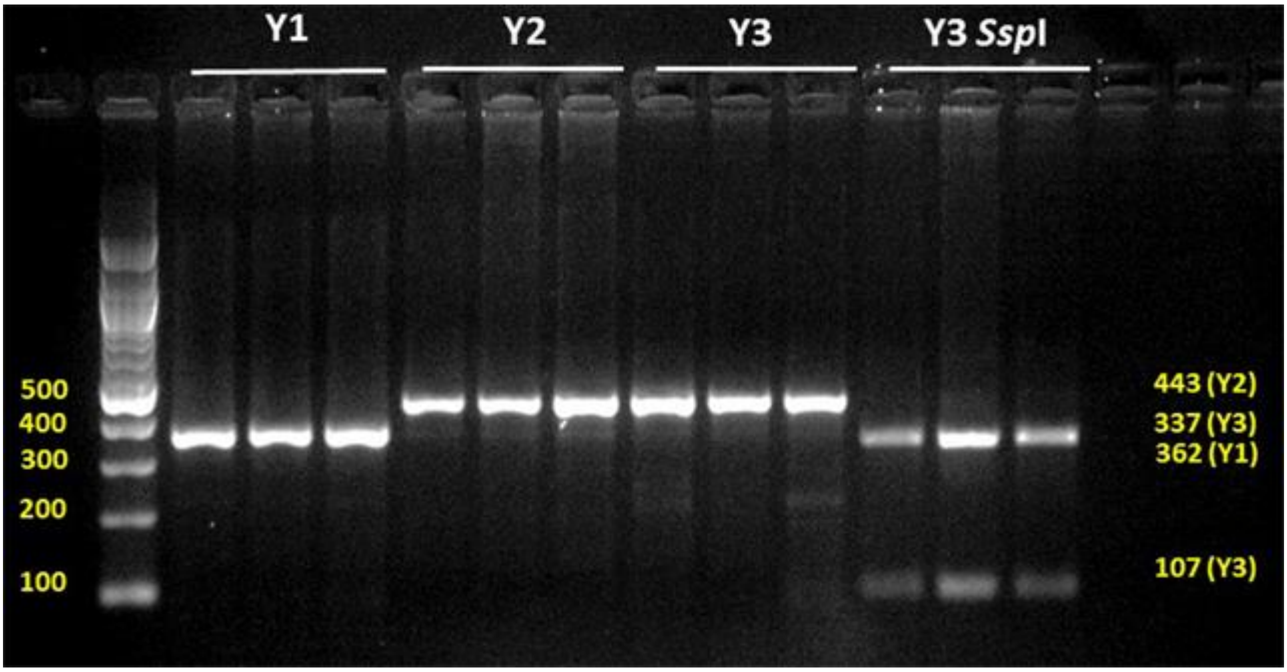

Supplementary Fig. S2: Analysis of USP9Y marker to differentiate Y1, Y2 and Y3 haplogroups using *Ssp* I PCR-RFLP. Y2 and Y3 display a fragment of 443 bp, while Y1 generates a fragment of 362 bp after PCR amplification. Upon digestion of 443 bp amplicon with *Ssp* I, Y3 yields two fragments of 337 bp and 107 bp due to the presence of variant cutting site for *Ssp* I, while Y2 yields a single fragment of 443 bp due to absence of cutting site for RE. The size marker is a 100 bp DNA ladder. DNA from HF, Jersey and Sahiwal bulls, already screened by allele-specific PCR and direct sequencing[7] with lineages Y1, Y2 and Y3 were used for the analysis of the USP9Y marker.

a.

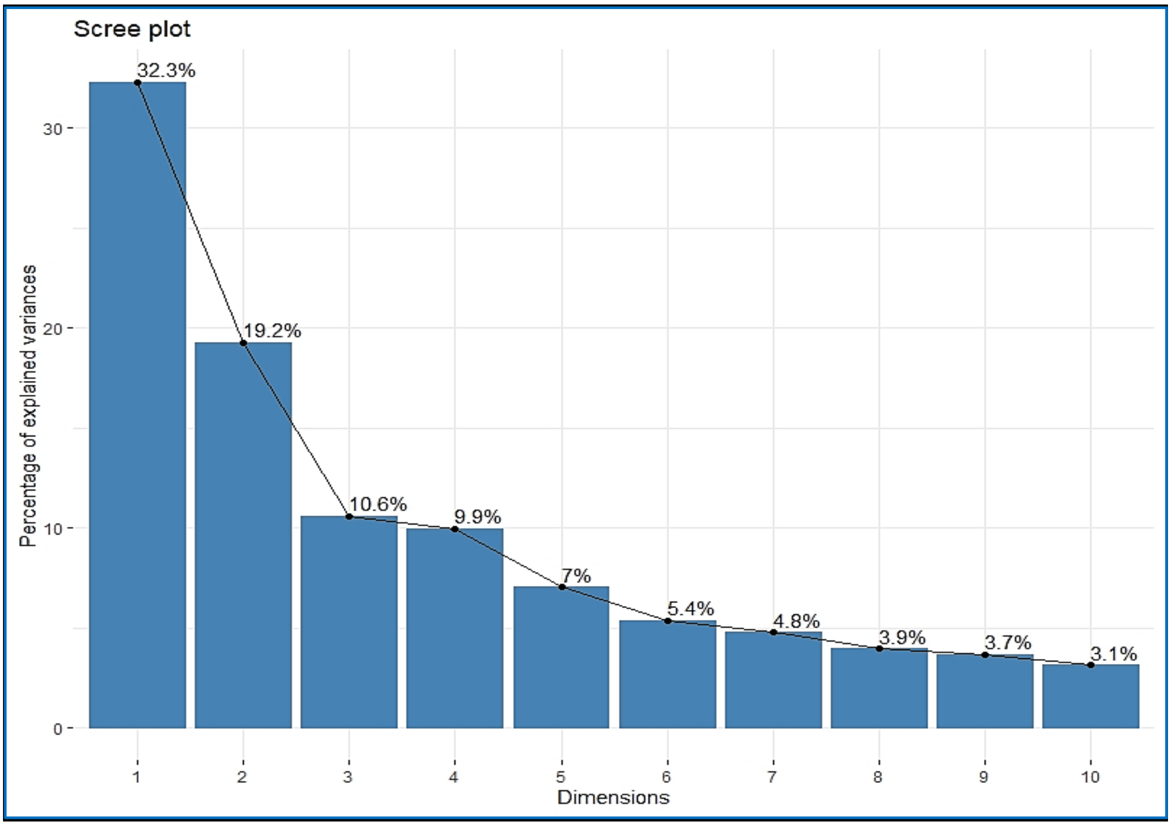
Scree plot
32.3%
19.2%
10.6%
9.9%
7%
5.4%
4.8%
3.9%
3.7%
3.1%
Percentage of explained variances
0
10
20
30
1
2
3
4
5
6
7
8
9
10
Dimensions

b

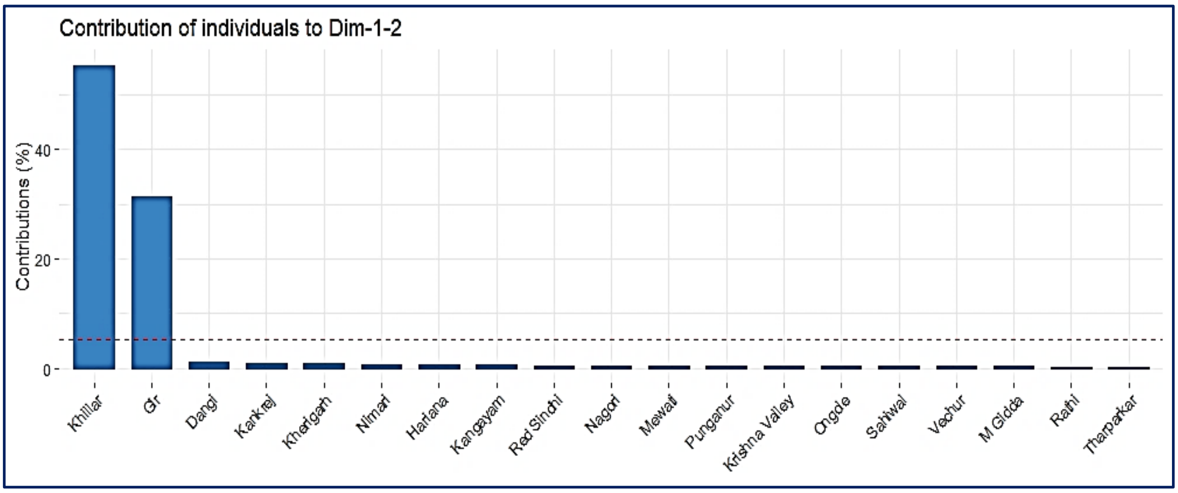
Contribution of individuals to Dim-1-2
Contributions (%)
0
20
40
Khillar
Gir
Dangi
Kankrej
Kherigarh
Nimari
Hariana
Kangayam
Red Sindhi
Nagori
Mewati
Punganur
Krishna Valley
Ongole
Sahiwal
Vechur
M Gidda
Rathi
Tharparkar

c.

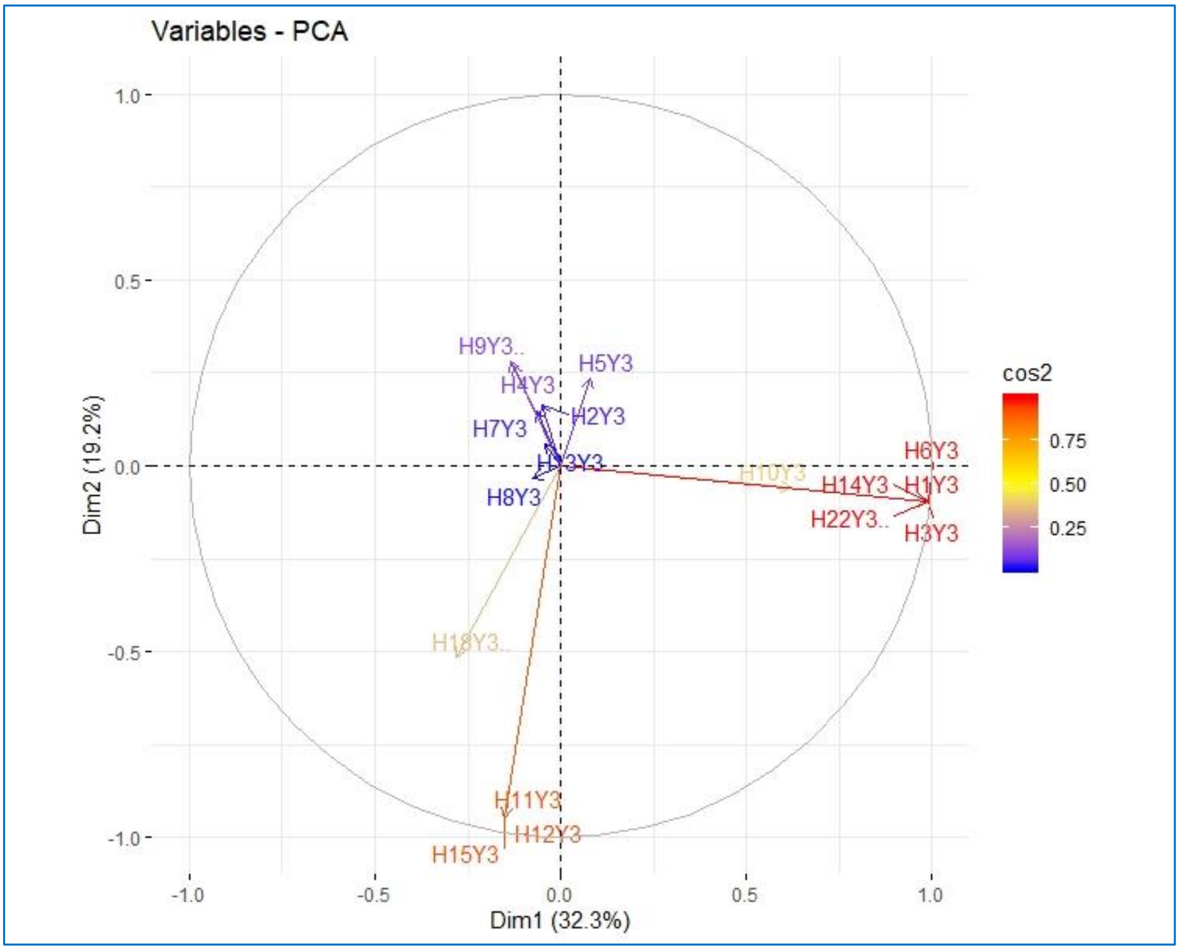

Variables - PCA
cos2
0.75
0.50
0.25
H9Y3..
H5Y3
H4Y3
H2Y3
H7Y3
H13Y3
H8Y3
H10Y3
H6Y3
H14Y3
H1Y3
H22Y3..
H3Y3
H18Y3..
H11Y3
H12Y3
H15Y3
Dim1 (32.3%)
Dim2 (19.2%)

d.

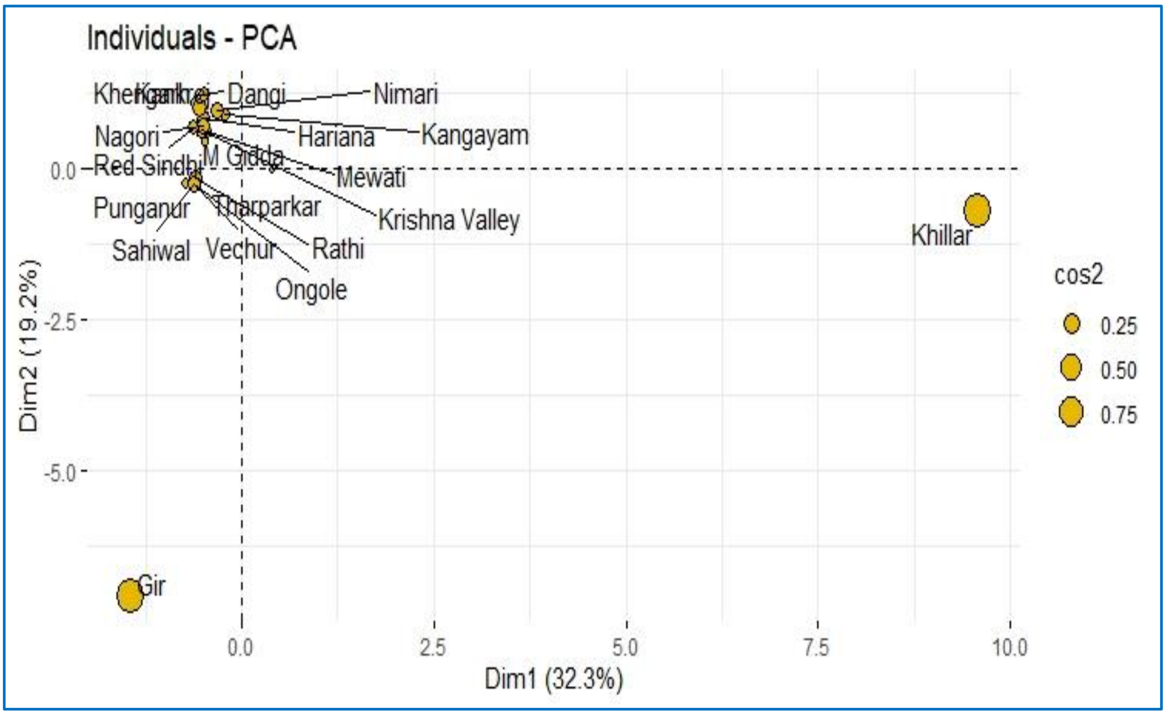

Individuals - PCA
Dangi
Nimari
Nagori
Hariana
Kangayam
Red-Sindhi
M Gidda
Mewati
Punganur
Tharparkar
Krishna Valley
Sahiwal
Vechur
Rathi
Ongole
Khillar
Gir
cos2
0.25
0.50
0.75
Dim1 (32.3%)
Dim2 (19.2%)

e.

	**Eigen value**	Variance percent	Cumulative variance percent
Dim.1	5.49E+00	3.23E+01	32.27921
Dim.2	3.27E+00	1.92E+01	51.5286
Dim.3	1.80E+00	1.06E+01	62.12532
Dim.4	1.69E+00	9.94E+00	72.07015
Dim.5	1.20E+00	7.03E+00	79.10116
Dim.6	9.12E-01	5.36E+00	84.4645
Dim.7	8.12E-01	4.77E+00	89.23863
Dim.8	6.71E-01	3.95E+00	93.18647
Dim.9	6.25E-01	3.68E+00	96.86374
Dim.10	5.33E-01	3.14E+00	100

Supplementary Fig. S3: PCA analysis based on Y-chromosome haplotype frequencies of all breeds. a. Contribution of PCs; b. Contribution of individuals (breeds) to dimension 1 and 2; c. Contribution of haplotypes; d. Contribution of breeds; e. Eigen values; M Gidda: Malnad Gidda.

a.

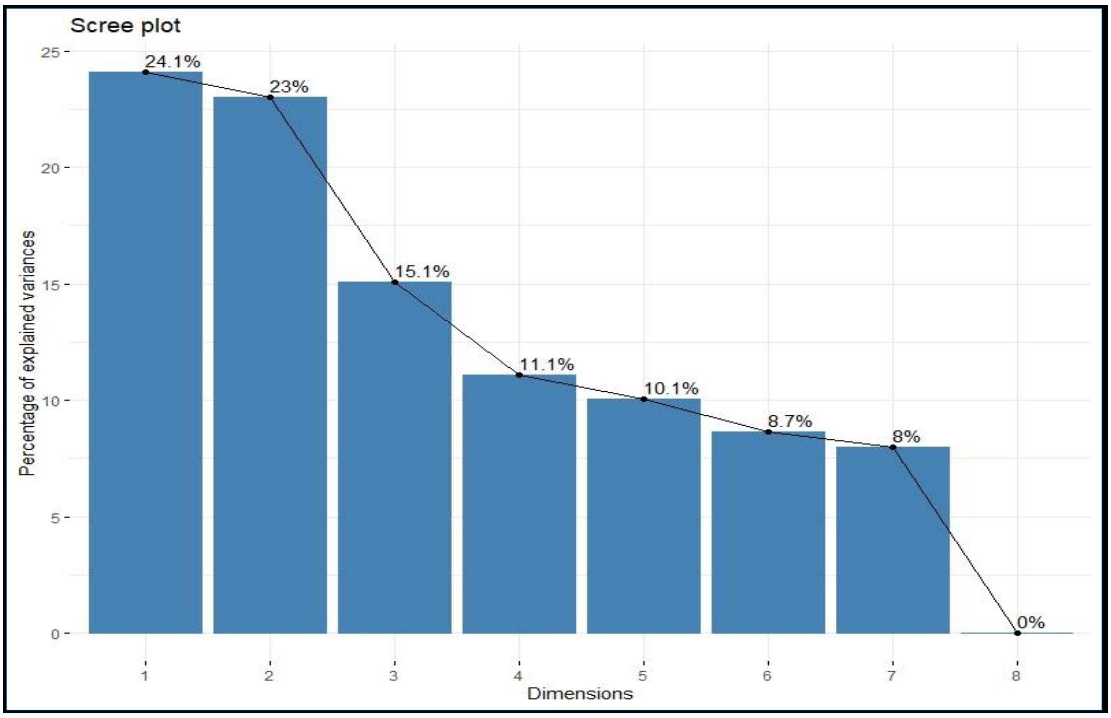
Scree plot
24.1%
23%
15.1%
11.1%
10.1%
8.7%
8%
0%
Percentage of explained variances
Dimensions

b

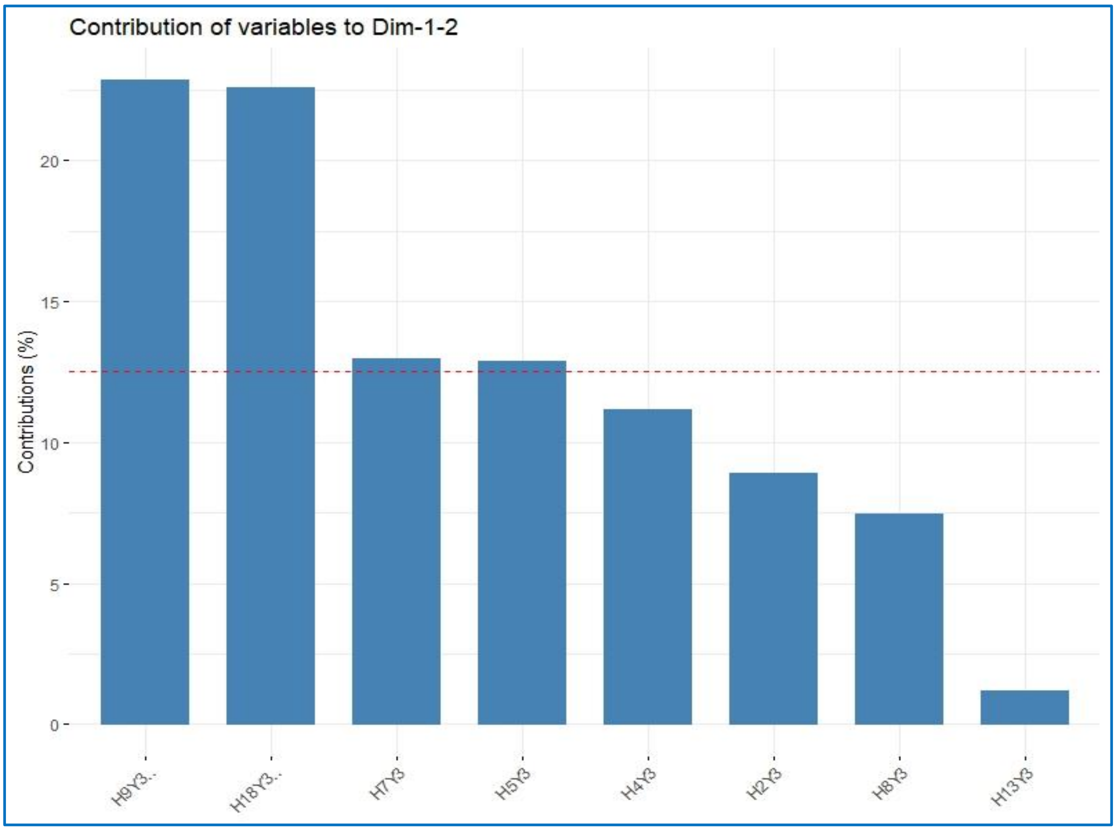
Contribution of variables to Dim-1-2
Contributions (%)
H9Y3..
H18Y3..
H7Y3
H5Y3
H4Y3
H2Y3
H8Y3
H13Y3

c.

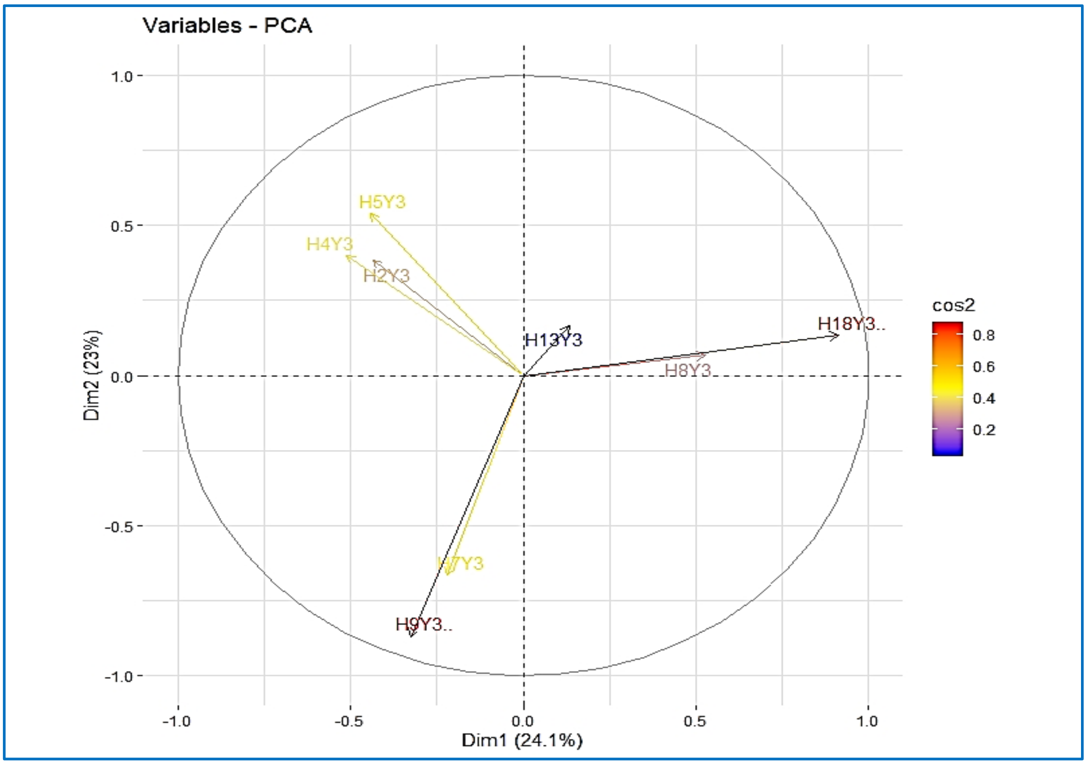
Variables - PCA
1.0
0.5
0.0
-0.5
-1.0
Dim2 (23%)
-1.0
-0.5
0.0
0.5
1.0
Dim1 (24.1%)
H5Y3
H4Y3
H2Y3
H13Y3
H18Y3..
H8Y3
H7Y3
H9Y3..
cos2
0.8
0.6
0.4
0.2

d.

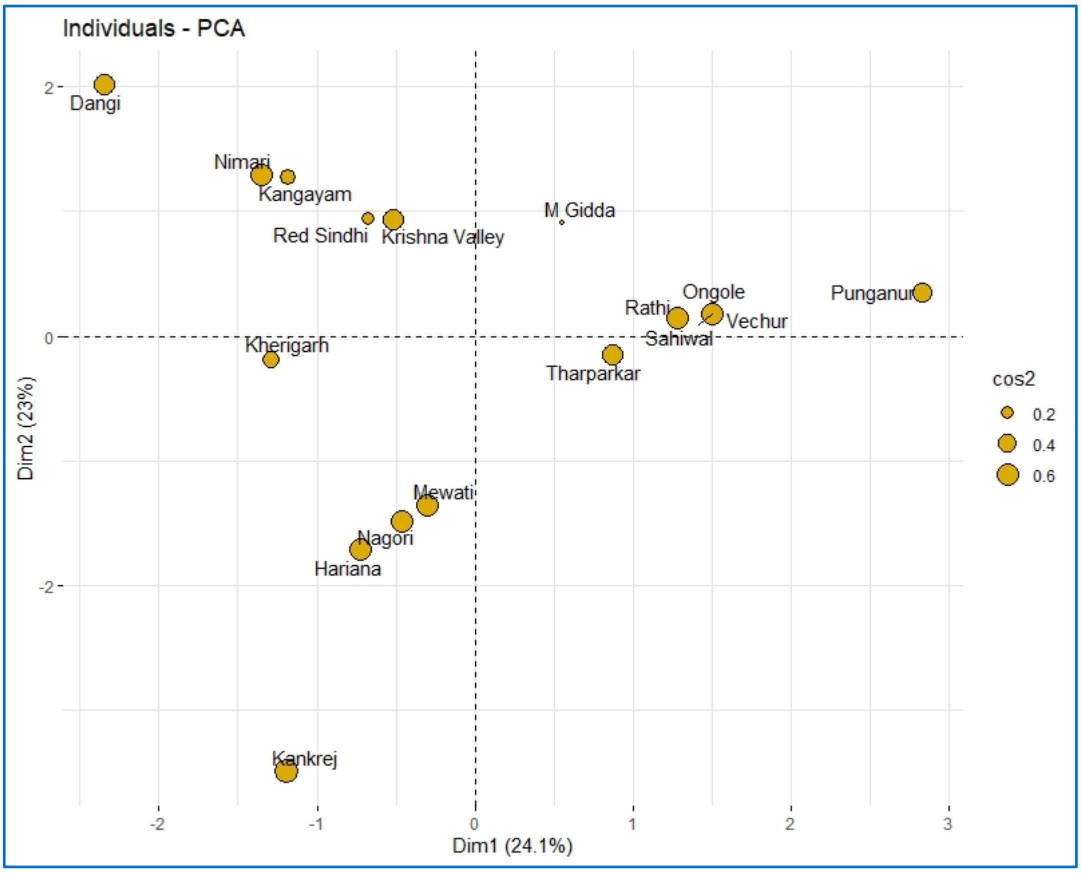
Individuals - PCA
2
0
-2
Dim2 (23%)
-2
-1
0
1
2
3
Dim1 (24.1%)
Dangi
Nimari
Kangayam
Red Sindhi
Krishna Valley
M Gidda
Ongole
Rathi
Punganur
Vechur
Sahiwal
Kherigarh
Tharparkar
Mewati
Nagori
Hariana
Kankrej
cos2
0.2
0.4
0.6

e.

	**Eigen value**	Variance percent	Cumulative variance percent
Dim.1	1.927892	24.09866	24.09866
Dim.2	1.840743	23.00929	47.10794
Dim.3	1.2058	15.0725	62.18045
Dim.4	0.886796	11.08495	73.2654
Dim.5	0.80549	10.06863	83.33402
Dim.6	0.692652	8.658148	91.99217
Dim.7	0.640018	8.000221	99.99239
Dim.8	0.000609	0.007607	100

Supplementary Fig. S4: PCA analysis based on Y-chromosome haplotype frequencies of remaining breeds after removal of Gir and Khillar. a. Contribution of PCs; b. Contribution of individuals (breeds) to Dimension 1 and 2; c. Contribution of haplotypes; d. Contribution of breeds; e. Eigen values; M Gidda: Malnad Gidda.

Online Supplementary References

1. Kappes, S. M. *et al.* A second-generation linkage map of the bovine genome. *Genome Res.* **7**, 235–249 (1997).
2. Bishop, M. D. *et al.* A genetic linkage map for cattle. *Genetics* **136**, 619–639 (1994).
3. Götherström, A. *et al.* Cattle domestication in the Near East was followed by hybridization with aurochs bulls in Europe. *Proc. Biol. Sci.* **272**(1579), 2345–2350 (2005).
4. Liu, W. S. *et al.* Bovine Y chromosome microsatellite polymorphisms. *Cytogenet. Genome. Res.* **102**(1-4), 53–58 (2003).
5. Ginja, C. *et al.* The genetic ancestry of American creole cattle inferred from uniparental and autosomal genetic markers. *Sci. Rep.* **9**, 1–16 (2019).
6. Ginja, C. *et al.* Origins and genetic diversity of New World Creole cattle: Inferences from mitochondrial and Y chromosome polymorphisms. *Anim. Genet.* **41**(2), 128–141 (2010).
7. Kumar, S. *et al.* Y-chromosome variation in Indian native cattle breeds and crossbred population. *Indian J. Anim. Res.* **51**, 1–7 (2017).
